# High-throughput microarray reveals the epitranscriptome-wide landscape of m^6^A-modified circRNA in oral squamous cell carcinoma

**DOI:** 10.1186/s12864-022-08806-z

**Published:** 2022-08-23

**Authors:** Wei Zhao, Jingwen Liu, Jie Wu, Xiaozhou Ma, Xi Wang, Leyu Zhang, Zhe Han, Jianming Yang, Yameng Cui, Xin Hu, Jiayin Deng

**Affiliations:** 1 The School and Hospital of Stomatology, Tianjin Medical University, 300070 Tianjin, China; 2 Institute of Orthopaedics, Tianjin Hospital, Tianjin, China; 3 Department of Immunology, Key Laboratory of Immune Microenvironment and Disease of the Educational Ministry of China, Tianjin Key Laboratory of Cellular and Molecular Immunology, School of Basic Medical Sciences, Tianjin Medical University, 300060 Tianjin, China; 4 Department of Integrated Traditional & Western Medicine, Tianjin Medical University Cancer Institute and Hospital, National Clinical Research Center for Cancer, 300060 Tianjin, China

**Keywords:** N^6^-Methyladenosine, Circular RNA, m^6^A-circRNAs, Oral squamous cell carcinoma, MeRIP-Seq.

## Abstract

**Background:**

Emerging transcriptome-wide high-throughput screenings reveal the landscape and functions of RNAs, such as circular RNAs (circRNAs), in human cancer. In addition, the post-transcriptional RNA internal modifications, especially N^6^-methyladenosine (m^6^A), greatly enrich the variety of RNAs metabolism. However, the m^6^A modification on circRNAs has yet to be addressed.

**Results:**

Here, we report an epitranscriptome-wide mapping of m^6^A-modified circRNAs (m^6^A-circRNA) in oral squamous cell carcinoma (OSCC). Utilizing the data of m^6^A methylated RNA immunoprecipitation sequencing (MeRIP-seq) and m^6^A-circRNAs microarray, we found that m^6^A-circRNAs exhibited particular modification styles in OSCC, which was independent of m^6^A-mRNA. Besides, m^6^A modification on circRNAs frequently occurred on the long exons in the front part of the coding sequence (CDS), which was distinct from m^6^A-mRNA that in 3’-UTR or stop codon.

**Conclusion:**

In conclusion, our work preliminarily demonstrates the traits of m^6^A-circRNAs, which may bring enlighten for the roles of m^6^A-circRNAs in OSCC.

**Supplementary Information:**

The online version contains supplementary material available at 10.1186/s12864-022-08806-z.

## Highlights

1. m^6^A-circRNAs exhibited their particular modification style in OSCC, which was independent of m^6^A-mRNA.

2. m^6^A on circRNAs frequently occurred on the long exons in the front part of CDS, which was distinct from m^6^A-mRNA that in 3’-UTR or stop codon.

**Supplementary Information:**

The online version contains supplementary material available at 10.1186/s12864-022-08806-z.

## Background

Oral squamous cell carcinoma (OSCC) acts as the most common cancers in head and neck, accounting for 90% of oral malignant tumor [1]. In addition to local hyperplasia, tissues erosion and dysfunction, OSCC frequently results in lymphatic metastasis to the neck area. Although comprehensive therapeutic schedules have made remarkable progress, including surgical excision, chemotherapy and radiotherapy, the survival and prognosis of OSCC sufferer remain poor [2]. The latest researches show that the tumorigenesis of OSCC is a complicated process involved in diverse alterations of genetic and epigenetic. Therefore, unremitting exploration targeting the initiation and oncogenesis of OSCC could provide valuable directions for precise treatment.

Circular RNAs (circRNAs) are groups of covalently closed loop transcripts in noncoding transcriptome [3]. CircRNAs are characterized by a continuous loop generated from the back-splicing of linear RNA, which is different from linear RNA. Benefited from covalently-bonded RNA molecule, circRNAs could resist the digestion of RNA enzyme and stably exist in the cellular microenvironment, which is responsible for their high abundancde in different species. In OSCC, the functions of circRNAs have been preliminarily uncovered. Certain circRNAs regulate the OSCC cellular glycolysis metabolism, e.g. circ_0000140 [4] and hsa_circRNA_100290 [5]. Certain circRNAs regulate the epithelial-mesenchymal transition (EMT) of OSCC, e.g. hsa_circ_0009128 [6] and circEPSTI1 [7]. Moreover, some circRNAs serve as potential diagnostic predictors or biomarkers for OSCC, e.g. hsa_circ_0008309 [8] and hsa_circ_0003829 [9]. Therefore, the evidence illustrates the critical roles participating in OSCC initiation and progression.

N^6^-methyladenosine (m^6^A) is one of the most abundant internal modifications in eukaryotic messenger RNA (mRNA) that exerts essential roles in mRNA fate, including mRNA stability, splicing and translation [10]. RNA m^6^A modification is a well-known chemical modification occurred in the sixth nitrogen. The specific transcriptome modifications have been proved to influence the cellular pathophysiology, thereby regulating tumorigenesis. For example, RNA modification enzyme methyltransferase-like 3 (METTL3) is upregulated in OSCC cohorts and the high expression of METTL3 is associated with poor prognosis. Functionally, METTL3 promotes cell proliferation, migration, invasion and self-renewal through promoting BMI1 translation under the cooperation with IGF2BP1 in OSCC [11]. Besides, m^6^A demethylase fat mass and obesity-associated protein (FTO) knockdown induces the downregulation of m^6^A-contained eIF4G1, which is captured by YTHDF2, and enhances the autophagic flux, thus inhibiting OSCC tumorigenesis. With the release of new research work about m^6^A, there are reasons to believe that the mechanism by which m^6^A regulates OSCC could be identified.

Given that m^6^A modification regulates the fate of RNA, while circRNAs are a group of covalent closed-loop RNA, there could be a pivotal connection between m^6^A and circRNAs [12]. In fact, current researches prove this hypothesis in human cancers. However, the role of m^6^A on the function of circRNAs has yet to be addressed. Here, our team utilized the data of MeRIP-seq and m^6^A-circRNAs epitranscriptomic microarray analysis to investigate the epitranscriptome-wide mapping of m^6^A-modified circRNA in OSCC. Our work focused on the traits of m^6^A-circRNAs and probed into the association of m^6^A-circRNAs and m^6^A-mRNA, which may bring enlighten for the roles of m^6^A-circRNAs in OSCC.

## Results

### m^6^A-circRNA epitranscriptomic microarray analysis revealed the m^6^A-circRNAs profile in OSCC

**Fig. 1:**
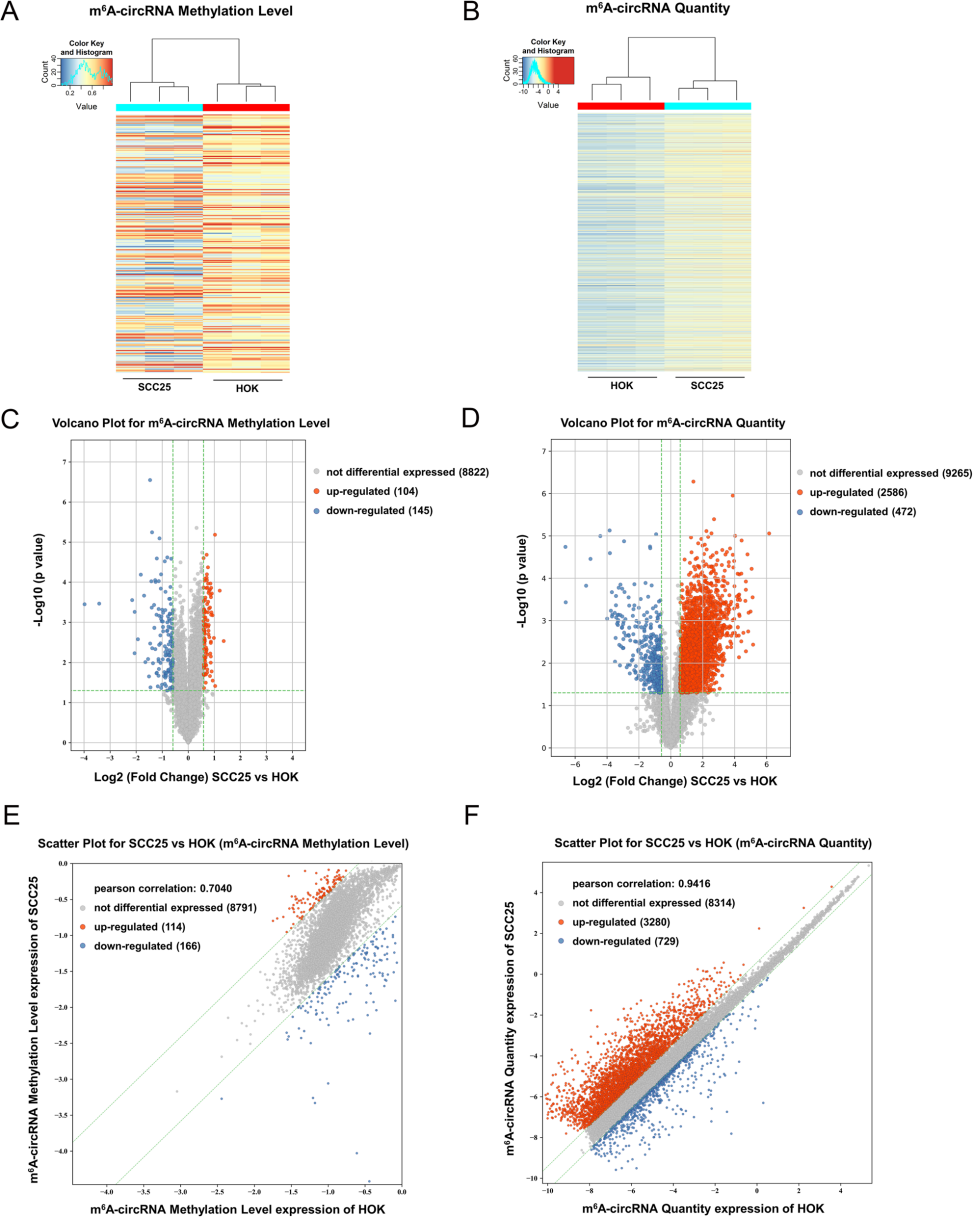
m^6^A-circRNA epitranscriptomic microarray analysis revealed the m^6^A- circRNAs profile in OSCC. **A**, **B** Heat Map showed the differentially expressed m^6^A-circRNAs, including ‘m^6^A-circRNA Methylation Level’ and ‘m^6^A-circRNA Quantity’. OSCC cells were SCC25 cells and normal cells were HOK cells. **C**, **D** Volcano Plot showed the differentially expressed m^6^A-circRNAs. Based on this threshold (*p*-value < 0.05, fold change > 1.5 fold), there were 104 up-regulated m^6^A-circRNA and 145 down-regulated m^6^A-circRNA on the basis of ‘m^6^A-circRNA Methylation Level’ (**C**). And, there were 2586 up-regulated m^6^A-circRNA and 472 down-regulated m^6^A-circRNA on the basis of ‘m^6^A-circRNA Quantity’ (**D**). **E**, **F** Scatter Plot displayed the correlation distribution of m^6^A-circRNA in SCC25 cells and HOK cells

m^6^A-circRNA epitranscriptomic microarray analysis was performed according to the workflow using SCC25 cells and HOK cells. As regarding to the microarray raw data, the differentially m^6^A-methylated circRNAs were expressed as ‘m^6^A methylation level’ and ‘m^6^A quantity’, which was different from conventional circRNA microarray analysis. Thus, in our m^6^A-circRNA epitranscriptomic microarray analysis, the differentially expressed m^6^A-circRNAs were presented by two aspects, including ‘m^6^A-circRNA Methylation Level’ and ‘m^6^A-circRNA Quantity’. The screening threshold was set to *p*-value < 0.05 and fold change > 1.5 fold. Based on this threshold, there were 104 up-regulated m^6^A-circRNA and 145 down-regulated m^6^A-circRNA on the basis of ‘m^6^A-circRNA Methylation Level’, meanwhile, there were 2586 up-regulated m^6^A-circRNA and 472 down-regulated m^6^A-circRNA on the basis of ‘m^6^A-circRNA Quantity’. The raw data of ‘m^6^A-circRNA Methylation Level’ and ‘m^6^A-circRNA Quantity’ were presented by Heat Map (Fig. 1A, B), Volcano Plot (Fig. 1C, D), and Scatter Plot t (Fig. 1E F). Taken together, m^6^A-circRNA epitranscriptomic microarray analysis revealed the m^6^A-circRNAs profile in OSCC.

### The significantly expressed m^6^A-circRNAs

**Fig. 2:**
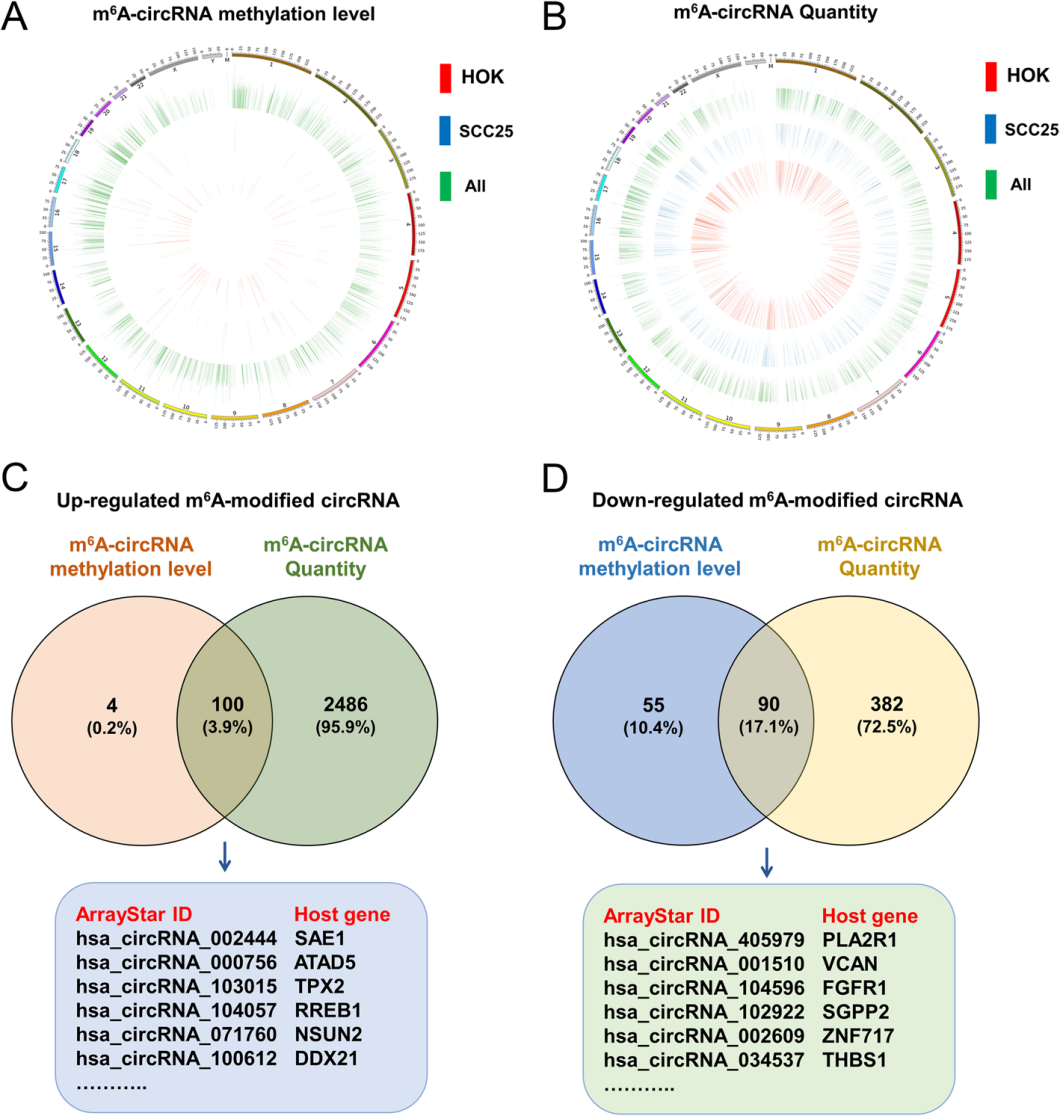
The significantly expressed m^6^A-circRNAs. **A**, **B** Circos plot showed the locations of m^6^A-cricRNAs on human chromosomes, including ‘m^6^A methylation level’ or ‘m^6^A quantity’. The outermost layer was chromosome map of human genome. The inner green layer indicated all the m^6^A-cricRNAs detected by m^6^A-circRNA epitranscriptomic microarray. The inner blue layer indicated the m^6^A-cricRNAs from OSCC cells (SCC25). The innermost red layer indicated the m^6^A-cricRNAs from normal control cells (HOK) cells. **C**, **D** Venn diagram showed the up-regulated 100 m^6^A-circRNAs and 90 down-regulated m^6^A-circRNAs. Several up-regulated or down-regulated m^6^A-circRNAs were randomly chosen and listed in the diagram

Given that m^6^A-circRNA epitranscriptomic microarray analysis discovered hundreds or thousands of circRNAs with different ‘m^6^A methylation level’ or ‘m^6^A quantity’, further analysis focused on their intersection to determine the potential m^6^A-methylated circRNAs with significant expression. Circos plot showed the locations of m^6^A-cricRNAs on human chromosomes, including ‘m^6^A methylation level’ or ‘m^6^A quantity’(Fig. 2A and B). In the schematic, the outermost layer was chromosome map of human genome. The inner green layer indicated all the m^6^A-cricRNAs detected by m^6^A-circRNA epitranscriptomic microarray. The inner blue layer indicated the m^6^A-cricRNAs from OSCC cells (SCC25), and the innermost red layer indicated the m^6^A-cricRNAs from normal control cells (HOK) cells.

In consideration of the fact that m^6^A-modified circRNAs were categorized and detected based on ‘m^6^A methylation level’ and ‘m^6^A quantity’, our team took the intersection of ‘m^6^A methylation level’ and ‘m^6^A quantity’ to zoom out the scope, thereby ascertaining the up-regulated or down-regulated m^6^A-circRNAs. Venn diagram showed the up-regulated m^6^A-circRNAs (100) (Fig. 2C) and down-regulated m^6^A-circRNAs (90) (Fig. 2D). Besides, several up-regulated or down-regulated m^6^A-circRNAs were randomly chosen and listed in the diagram. Moreover, among these screened m^6^A-circRNAs, the significantly up-regulated (Top 10) m^6^A-circRNAs were shown in Table 1 and down-regulated m^6^A-circRNAs were shown in Table 2.

**Table 1:** Top 10 up-regulated m^6^A-circRNAs in OSCC

dysregulated	ArrayStar ID	circBase ID	*p*-value	Log_2_FC	Host gene	Chromosome	Length (bp)	Location
Up	hsa_circRNA_026358	hsa_circ_0026358	6.559E-06	2.037	KRT7	chr12	534	52,628,938–52,635,420
Up	hsa_circRNA_025042	hsa_circ_0025042	4.266E-05	1.688	FOXM1	chr12	549	2,983,142–2,983,691
Up	hsa_circRNA_101929	hsa_circ_0004931	6.379E-05	1.642	NXN	chr17	460	722,678–729,318
Up	hsa_circRNA_000756	hsa_circ_0000756	8.923E-05	1.644	ATAD5	chr17	1366	29,170,930–29,196,664
Up	hsa_circRNA_007923	hsa_circ_0007923	7.750E-05	1.632	RRM1	chr11	631	4,123,222–4,133,292
Up	hsa_circRNA_101081	hsa_circ_0000408	2.511E-05	1.503	TIMELESS	chr12	378	56,824,664–56,826,308
Up	hsa_circRNA_104564	hsa_circ_0083444	8.186E-05	1.549	MTUS1	8hr8	2441	17,601,112–17,613,470
Up	hsa_circRNA_103069	hsa_circ_0060516	2.781E-04	1.560	PABPC1L	chr20	235	43,547,546–43,547,918
Up	hsa_circRNA_407080	None	4.701E-03	1.629	CHD7	chr8	-	61,707,544–61,714,152
Up	hsa_circRNA_403287	None	9.186E-05	1.544	LPCAT1	chr5	-	1,488,505–1,501,718

**Table 2:** Top 10 down-regulated m^6^A-circRNAs in OSCC

dysregulated	circRNA	circBase ID	*p*-value	Log_2_FC	Host gene	Chromosome	Length	Location
Down	hsa_circRNA_001819	hsa_circ_0000658	2.829E-07	0.362	MCTP2	chr15	1502	94,847,149–94,848,651
Down	hsa_circRNA_104898	hsa_circ_0088249	5.702E-06	0.381	PAPPA	chr9	994	119,106,821–119,130,033
Down	hsa_circRNA_001484	hsa_circ_0000701	8.072E-06	0.465	CHD9	chr16	3095	53,188,358–53,191,453
Down	hsa_circRNA_000094	hsa_circ_0000247	9.871E-05	0.025	MCU	chr10	792	74,474,868–74,475,660
Down	hsa_circRNA_101122	hsa_circ_0027842	6.493E-05	0.283	ANKS1B	chr12	535	100,200,181–100,219,167
Down	hsa_circRNA_008421	hsa_circ_0008421	9.522E-05	0.374	FBN1	chr15	374	48,888,479–48,905,289
Down	hsa_circRNA_000403	hsa_circ_0000403	2.563E-05	0.433	ZNF385A	chr12	80	54,764,057–54,764,137
Down	hsa_circRNA_092561	hsa_circ_0001612	2.605E-05	0.636	SENP6	chr6	498	76,331,247–76,357,517
Down	hsa_circRNA_022382	hsa_circ_0022382	9.482E-04	0.493	FADS2	chr11	411	61,605,249–61,608,197
Down	hsa_circRNA_008786	hsa_circ_0008786	8.307 E-04	0.485	POLE2	chr14	424	50,120,707–50,122,529

### The genomic characteristic of m^6^A-circRNAs in OSCC

**Fig. 3:**
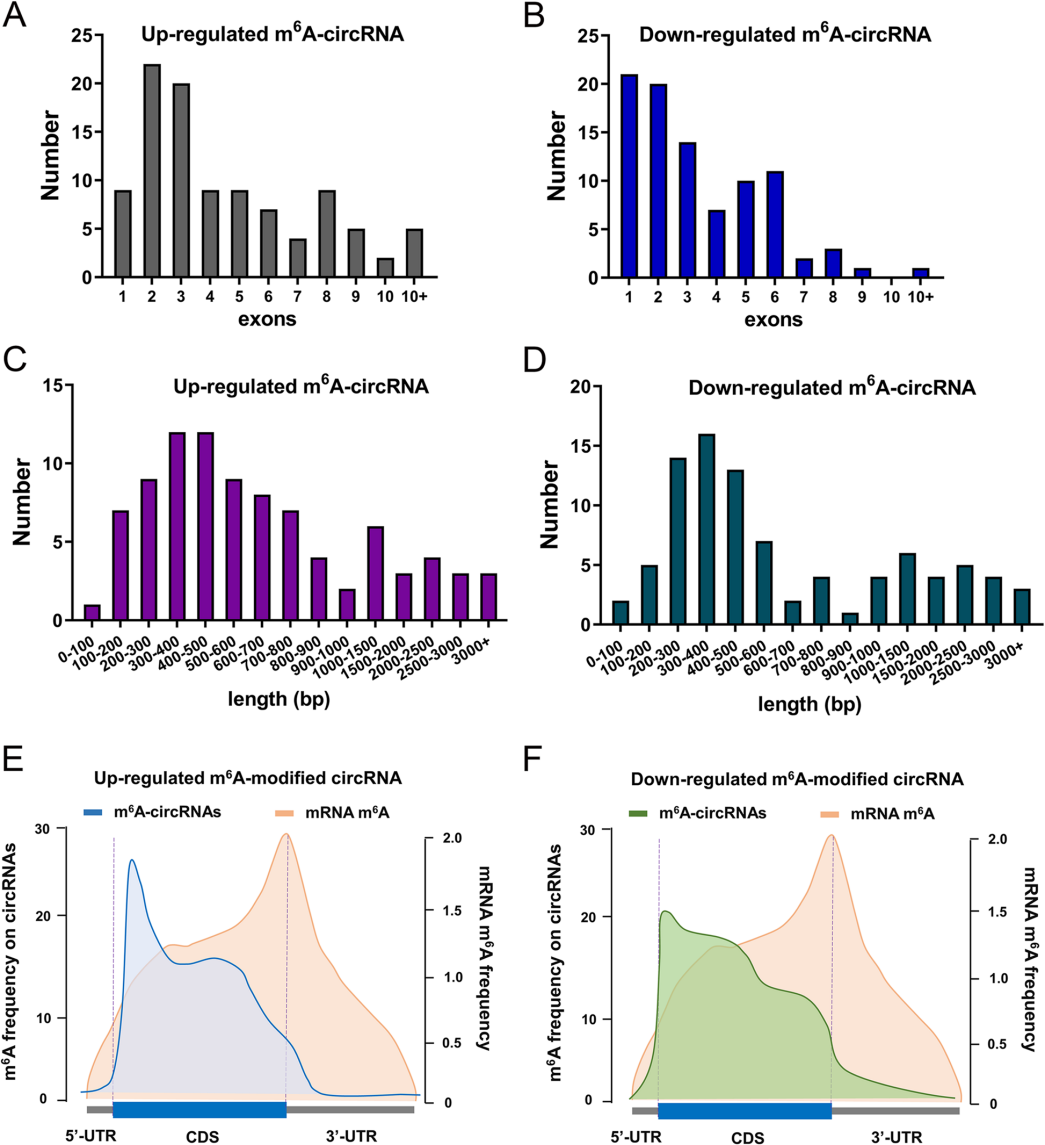
The genomic characteristic of m^6^A-circRNAs in OSCC. **A**, **B** The exons quantity of up-regulated and down-regulated m^6^A-modified circRNAs were counted in differentially expressed m^6^A-modified circRNAs, including up-regulated (100 m^6^A-circRNAs) and down-regulated (90 m^6^A-circRNAs). **C**, **D** The length (spliced length, not genomic length) was counted by stages count (~ 100 bp). **E**, **F** Metagene profiles of enrichment of m^6^A modification sites on mRNA and circRNA of gene locus across RNA transcriptome. 5’-untranslated regions (5’-UTR), coding sequences (CDS), 3’-untranslated regions (3’-UTR)

An essential literature by Dr. Alan C Mullen [13] (2017) has raised an enlightening point that m^6^A-modified circRNAs exhibit characteristic rules, thus, we investigate the genomic characteristic of m^6^A-circRNAs in OSCC with the help of MeRIP-Seq and m^6^A-circRNA epitranscriptomic microarray. The differentially expressed m^6^A-modified circRNAs, including up-regulated (100 m^6^A-circRNAs) and down-regulated (90 m^6^A-circRNAs), were incorporated into the research. Firstly, exons quantity of up-regulated (Fig. 3A) and down-regulated (Fig. 3B) m^6^A-modified circRNAs were counted. Results illustrated that the exons of m^6^A-circRNAs mainly concentrated on 1–3 exons. Moreover, the length (spliced length, not genomic length) was counted by stages (100 bp). Results indicated that the length of m^6^A-circRNAs primarily distributed at 200–500 bp (Fig. 3C, D). In other words, the shorter circRNAs with a small number of exons were more easily methylated. Our previous research performed MeRIP-seq to detect the m^6^A profile in OSCC cells [14]. Taking MeRIP-seq as reference, we could find the difference and connection between m^6^A-mRNA and m^6^A-circRNAs. Metagene profiles showed the enrichment of m^6^A modification on mRNA and circRNA across RNA transcriptome. In up-regulated (Fig. 3E) and down-regulated (Fig. 3F) m^6^A-circRNAs, the m^6^A modification sites were primarily located in the front part of CDS, which was distinct from m^6^A-mRNA that in 3’-UTR or stop codon. Taken together, the data of m^6^A-circRNA epitranscriptomic microarray revealed the genomic characteristic of m^6^A-circRNAs in OSCC.

### The association of m^6^A-circRNAs and m^6^A-mRNA in OSCC

**Fig. 4:**
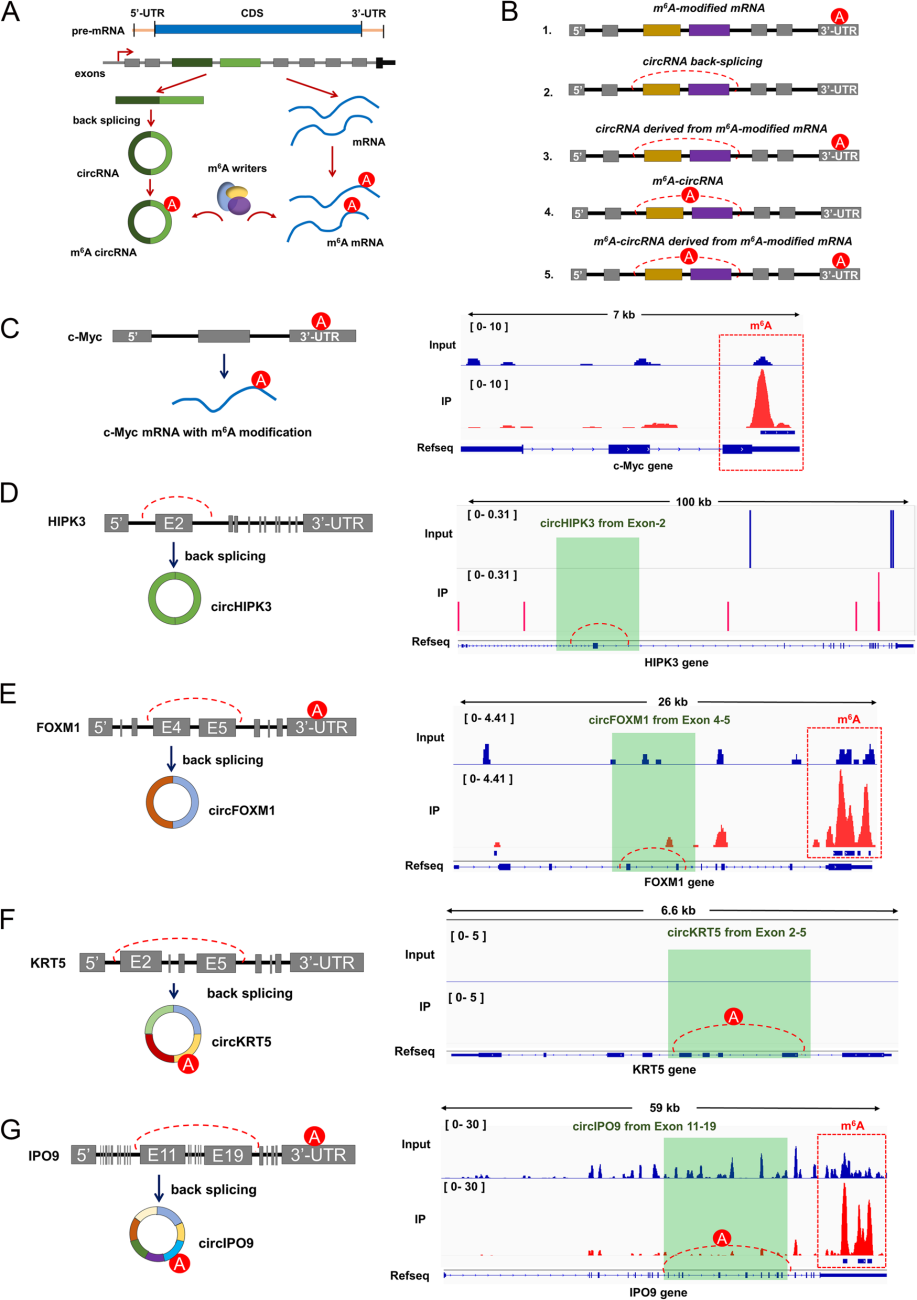
The association of m^6^A-circRNAs and m^6^A-mRNA in OSCC. **A** Schematic diagram illustrated the biogenesis of m^6^A-circRNAs and m^6^A-mRNA in OSCC cells. The m^6^A modification was installed by identical m^6^A methyltransferase complex (m^6^A writers). Red A indicated the m^6^A modification site. **B** Schematic diagram presented the genomic structure of m^6^A-mRNA, unmethylated circRNAs and m^6^A-circRNAs. **C** c-Myc mRNA acted as a representative for m^6^A-mRNA, which was methylated at 3’-UTR and without circRNA derived. **D** circHIPK3 (hsa_circ_0000284) represented the group of circRNAs without m^6^A modification in neither circular transcripts nor host gene. **E** CircFOXM1 (hsa_circ_0025039) represented the group of circRNAs without m^6^A modification in circRNA transcript, however, their host genes were m^6^A-modified at 3’-UTR. **F** CircKRT5 (hsa_circ_0026457) represented the group of circRNAs only m^6^A-modified in circRNA transcript, instead of host genes. **G** CircIPO9 (hsa_circ_0015936) represented the group of circRNAs m^6^A-modified both in circRNA transcript and their host genes

To date, the major field of m^6^A epitranscriptome research focused on mRNA m^6^A modification. In previous published literature, our team had performed the MeRIP-seq on OSCC prior to present m^6^A-circRNAs epitranscriptomic microarray analysis [14]. Utilizing the MeRIP-seq data and m^6^A-circRNAs epitranscriptomic microarray data, we found several traits of m^6^A-circRNAs and probed into the association of m^6^A-circRNAs and m^6^A-mRNA in OSCC. Combined with the existing literature and our findings, we confirmed that exons of certain gene could generate both pre-mRNA transcripts with m^6^A modification and exon-derived circRNAs with m^6^A modification (Fig. 4A). Moreover, the m^6^A modification was installed by identical m^6^A methyltransferase complex. Indeed, there might be other types of m^6^A-modified circRNAs, e.g. intron-derived circRNAs or intron/exon-derived circRNAs, which were elusive and will be investigate in our further research.

All the profound analysis was performed based on our MeRIP-seq data and m^6^A-circRNAs epitranscriptomic microarray data. According to the difference of methylated location, we concluded several subgroups of m^6^A-modified circRNAs (Fig. 4B). Firstly, a representative non-circRNA-derived mRNA (c-Myc mRNA) with m^6^A modification was selected as negative control (Fig. 4C). The m^6^A modification site was located in the 3’-UTR of c-Myc mRNA. Besides, a circRNA-derived gene (HIPK3) was selected as positive control, whose exon-2 was cyclized to be circHIPK3 (hsa_circ_0000284) (Fig. 4D). Our MeRIP-seq data revealed that there wasn’t any remarkable m^6^A modified site in the HIPK3 mRNA. The subgroup of circRNA, such as circHIPK3, is a more common type of transcripts without m^6^A modification neither in circRNA nor host gene.

Furthermore, we investigated the interaction of m^6^A modification with circRNAs or their host genes. We noticed a feature that whether circRNAs were m^6^A-modified wasn’t related to their host genes methylated or not. For example, circFOXM1 (hsa_circ_0025039) was a non-m^6^A-modified circRNA, however, its host gene (FOXM1) was remarkably m^6^A-modified at 3’-UTR (Fig. 4E). In addition to the circRNAs derived from m^6^A-modified host genes, another group of m^6^A-modified circRNAs was cyclized from unmethylated host genes. For example, crcKRT5 (hsa_circ_0026457) was a m^6^A-modified circRNA, however, there wasn’t any m^6^A site in its host gene (KRT5) (Fig. 4F). Moreover, the m^6^A modification could be installed both circRNA transcript and host genes. For instance, circIPO9 (hsa_circ_0015936) was a m^6^A-modified circRNA, besides, its host gene (IPO9) was m^6^A-modified at 3’-UTR (Fig. 4G).

Overall, based on MeRIP-seq and m^6^A-circRNAs epitranscriptomic microarray data, our findings illustrated that m^6^A-circRNAs exhibited their particular modification style in OSCC, which was independent of m^6^A-mRNA.

### GO and KEGG pathway analysis

**Fig. 5:**
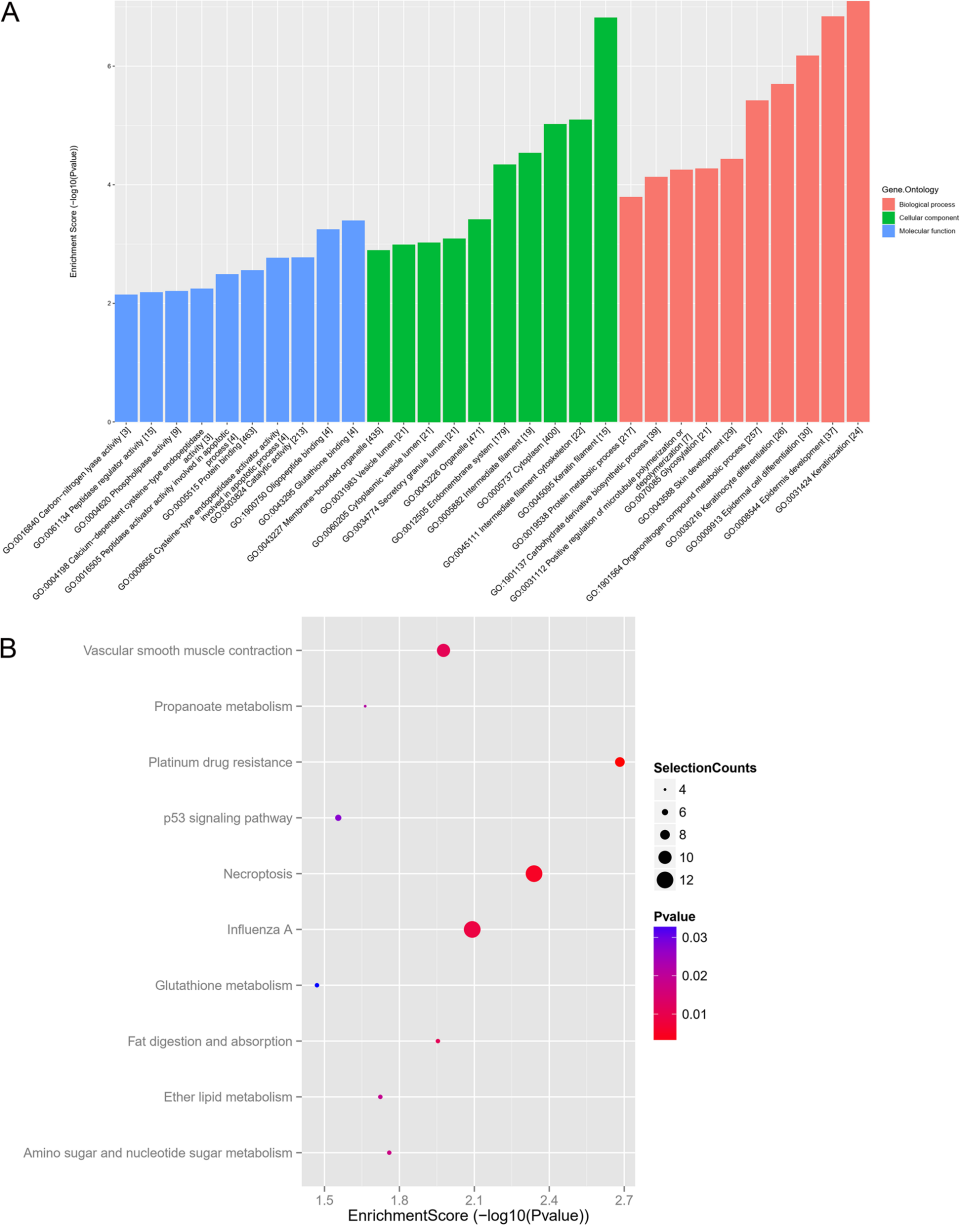
GO and KEGG Pathway Analysis. **A** Gene Ontology (GO) enrichment analysis of biological process (BP), cellular component (CC), and molecular function (MF). The vertical axis stands for the enrichment score of GO terms. **B** Bulb map displayed the top 10 KEGG pathways for host genes of differentially expressed circRNAs. The GO and KEGG analysis got permission of KEGG Database [15–17]. The size of the dot indicates the gene counts enriched in the pathway. The of the dot indicates the significance (*p* value) of the enriched pathway

To investigate the mechanisms correlated to m^6^A-circRNAs in OSCC, Gene Ontology (GO) enrichment analysis and Kyoto Encyclopedia of Genes and Genomes (KEGG) pathway analysis of the host genes of differentially expressed circRNAs were performed. GO terms included the biological process (BP), cellular component (CC), and molecular function (MF) categories. The top 10 enriched GO terms in BP, CC and MF were ‘keratinization’, ‘Keratin filament’ and ‘glutathione binding’ respectively (Fig. 5A). The top 10 KEGG pathways were shown as following (Fig. 5B) and the host genes of differentially expressed circRNAs were mainly associated with necroptosis. Given that circRNAs could act as miRNA sponge to harbor their downstream miRNAs, thereby releasing the fettered target mRNAs to modulate the cancer progression. The regulation pattern was regarded as competing endogenous RNA (ceRNA) (Supplementary Info: supplementary Figure S2).

## Discussion

As an indispensable part of epigenetic regulation, m^6^A modification is suggested as a crucial regulator participating in human cancer tumorigenesis. Chemical modification of m^6^A on RNAs is an efficient way of regulating molecular function, which influences the downstream pathways. Increased knowledge of m^6^A modification of noncoding RNA highlighted their effect on gene expression. Modifications on RNA contribute to the post-transcriptional regulation of RNA fate. Here, our research focused on the m^6^A-modified circRNA (m^6^A-circRNA) epitranscriptome-wide mapping in OSCC, which may provide valuable references for tumor epigenetics research.

CircRNAs have been identified to play important roles in tumorigenesis by virtue of their stability and enzyme-resistance. High-throughput sequencing for circRNAs reveals that many genes, previously considered as protein-coding genes, can produce circRNAs by back-splicing. In a variety of tumor subgroup, circRNAs participate in the cell differentiation, proliferation, energy metabolism and chemoradiotherapy resistance. As the research of circRNA progressed, the functions of circRNAs have been gradually investigated. The achievement is acquired not only in solid cancers, but also in OSCC. Our previous research found that, in OSCC, circUHRF1 up-regulated in OSCC and accelerated the tumorigenesis [18]. Up to now, the major regulatory type of circRNAs is competing endogenous RNA (ceRNA), by which circRNAs sponge micro RNAs to relieve the activity restrain of target proteins, e.g. circHIPK3 [19], circFOXO3 [20] and circIGHG [21]. In addition to ceRNA, more regulation manners of circRNAs have been reported. For instance, circ-ZNF609 is associated with heavy polysomes and could be translated into protein via splicing-dependent and cap-independent manner [22]. Thus, with the further research, the modes of circRNAs will be reported more and more.

Alan C. Mullen et al. (2017) firstly reported the profile of m^6^A modifications on circRNAs through a computational pipeline (AutoCirc) tool [13]. In this study, researchers defined thousands of m^6^A-circRNAs with cell-type-specific expression. Besides, m^6^A-circRNAs share identical m^6^A readers and writers with mRNAs, however, m^6^A-circRNAs are frequently derived from exons that unmethylated in mRNAs. Moreover, this study proposed another critical trait that the same exons methylated both on mRNA and m^6^A-circRNAs exhibit less stability. Overall, this groundbreaking work makes a valuable contribution to the field of m^6^A-modified circRNAs.

Here, our study performed the newely-developed m^6^A-circRNA epitranscriptomic microarray to detect the profile of m^6^A-modified circRNAs in OSCC. In our results, the differentially expressed m^6^A-circRNAs were presented by two aspects, including ‘m^6^A-circRNA Methylation Level’ and ‘m^6^A-circRNA Quantity’ based on threshold of *p*-value < 0.05 and fold change > 1.5 fold. Finally, there were 104 up-regulated m^6^A-circRNA and 145 down-regulated m^6^A-circRNA on the basis of ‘m^6^A-circRNA Methylation Level’, meanwhile, there were 2586 up-regulated m^6^A-circRNA and 472 down-regulated m^6^A-circRNA on the basis of ‘m^6^A-circRNA Quantity’. In previous study, our team had performed the MeRIP-seq on OSCC and thus we analyzed the genomic characteristic of m^6^A-circRNAs in OSCC utilizing the data of MeRIP-Seq and m^6^A-circRNA epitranscriptomic microarray. The differentially expressed m^6^A-modified circRNAs were selected into the research, including up-regulated (100 m^6^A-circRNAs) and down-regulated (90 m^6^A-circRNAs). The role of these up-regulated/down-regulated m^6^A-circRNAs is a promising research direction in OSCC progression. Combining with existing literature, the circRNAs with m^6^A modification display critical roles in tumor pression, which need more investigation.

As regarding to the genomic characteristic of m^6^A-circRNA in OSCC, we found that the exons of m^6^A-circRNAs mainly concentrated on 1–3 exons, and the length primarily distributed at 200–500 bp. Moreover, shorter circRNAs with a small number of exons were more easily methylated. Then, we analyzed the enrichment of m^6^A modification on mRNA and circRNA across RNA transcriptome. Results indicated that the m^6^A modification sites of m^6^A-circRNAs were primarily located in the front part of CDS, which was distinct from m^6^A-mRNA that in 3’-UTR or stop codon. Taken together, the genomic characteristic of m^6^A-circRNAs in OSCC was preliminarily investigated with the help of MeRIP-Seq and m^6^A-circRNA epitranscriptomic microarray.

To date, emerging literatures report the cross-talking of m^6^A and circRNA in human cancer [23]. Within the scope of epigenetics, RNA modifications and circRNAs are two rapidly expanding fields, and increasing number of researchers are beginning to turn their attention in this direction. For example, the m^6^A modification of circNSUN2 increases its export to the cytoplasm and circNSUN2 enhances the stability of HMGA2 mRNA to promote colorectal carcinoma metastasis progression, forming a circNSUN2/IGF2BP2/HMGA2 RNA-protein ternary complex [24]. In another example, METTL3 and YTHDC1 control the circ-ZNF609 accumulation and back-splicing reaction [25]. Moreover, in colorectal cancer, METTL3 induced the overexpression of circ1662 by binding the flanking sequences through installing its m^6^A modification [26]. In spite of the evidence showing essential roles of m^6^A and circRNA on human cancer, the comprehensive analysis of m^6^A-circRNA at epitranscriptome-wide is still absent. Actually, the direct evidence that in support of the function of m^6^A-circRNA are worth looking forward.

For the limitations in present, there was one OSCC cell line (SCC25) in the microarray, which is the limitations and insufficient for present research. Besides, despite this sequencing data of m^6^A-circRNA microarray and MeRIP-Seq, more cellular biochemistry experimental data are also needed.

## Conclusion

In conclusion, this research reveals the epitranscriptome-wide mapping of m^6^A-modified circRNA in OSCC. The findings expand our understanding of circRNAs and enrich the roles of m^6^A-circRNAs in OSCC, providing potential resolution strategy for OSCC targeted therapy.

## Materials and methods

### Human m^6^A-circRNA epitranscriptomic microarray analysis

The m^6^A-circRNA Epitranscriptomic microarray analysis was performed by Aksomics Inc (KangChen Bio-tech, Shanghai, China). Total RNA from cell samples (SCC25 cells, HNOK cells) was quantified using the NanoDrop ND-1000. The microarray hybridization was performed based on the Arraystar’s standard protocol. In brief, total RNAs were immunoprecipitated with anti-m^6^A rabbit polyclonal antibody (Synaptic Systems, 202,003). The unmodified RNAs were recovered from the supernatant as ‘Sup’. The m^6^A-modified RNAs were eluted from the immunoprecipitated magnetic beads as the ‘IP’. Then, ‘Sup’ and ‘IP’ RNAs were administrated with RNase R (Epicentre, Inc.), and subsequently labeled with Cy3 and Cy5 respectively as cRNAs s in separate reactions by Arraystar Super RNA Labeling Kit (Arraystar, AL-SE-005). The cRNAs were combined and hybridized on Arraystar Human circRNA Epitranscriptomic Microarray (8 × 15 K, Arraystar). Slides were washed and the arrays were scanned by an Agilent Scanner G2505C in two-color channels.

Array images were analyzed by Agilent Feature Extraction software (version 11.0.1.1). Raw intensities of Sup (Supernatant, Cy3-labelled) and IP (Immunoprecipitated, Cy5-labelled) were normalized with average of log2-scaled Spike-in RNA intensities. After Spike-in normalization, the probe signals having Present (P) or Marginal (M) QC flags in at least 3 out of 6 samples were retained for further ‘m^6^A methylation level’ and ‘m^6^A quantity’ analyses.

‘m^6^A methylation level’ was calculated for the percentage of modification based on the IP (Cy5-labelled) and Sup (Cy3-labelled) normalized intensities. ‘m^6^A quantity’ was calculated for the m^6^A methylation amount based on the IP (Cy5-labelled) normalized intensities. Differentially m^6^A-methylated circRNAs between two comparison groups were identified by filtering with the fold change and statistical significance (p-value) thresholds. Hierarchical Clustering was performed to show the distinguishable m^6^A-methylation pattern among samples.

### Workflow of m^6^A-circRNA epitranscriptomic microarray analysis

The workflow of human m^6^A-circRNA microarray analysis in OSCC cells was presented in the graphical representation (Figure S1), including RNA extraction, quality control (QC), library construction and data analysis. There were 3 pairs of samples for the m^6^A-circRNA epitranscriptomic microarray analysis, including OSCC cells (3 independent samples, SCC25 cells) and normal cells (3 independent samples, HOK cells). After the m^6^A immunoprecipitation, the m^6^A-modified RNAs were eluted from the immunoprecipitated magnetic beads were marked as ‘IP’ group (immunoprecipitated RNAs), and the unmodified RNAs were recovered from the supernatant and marked as ‘Sup’ group (supernatant unmodified RNAs) (Supplementary Info: supplementary Figure S1A). Regarding the raw data, the statistic analysis was performed from two aspects, including m^6^A methylation level and m^6^A quantity (Supplementary Info: supplementary Figure S1B). The data from m^6^A-circRNA epitranscriptomic microarray analysis reveals the landscape of m^6^A-modified circRNAs in OSCC.

### m^6^A-circRNA data analysis

The ‘m^6^A methylation level’ for a transcript was calculated as the percentage of modified RNA (modified %) in all RNAs based on the IP (Cy5-labelled) and Sup (Cy3-labelled) normalized intensities:

$$\text{modified}{\%}= \frac{\text{modified RNA}}{\text{Total RNA}} = \frac{\text{IP}}{\text{IP}+\text{Sup}}$$

$$= \frac{{\varvec{I}\varvec{P}}_{Cy5\;normalized\;intensity}}{{\varvec{I}\varvec{P}}_{Cy5\;normalized\;intensity}+{\varvec{S}\varvec{u}\varvec{p}}_{Cy3\;normalized\;intensity}}$$

Postscript: Raw intensities of IP (immunoprecipitated, Cy5-labelled) and Sup (supernatant, Cy3-labelled) were normalized with average of log2-scaled Spike-in RNA intensities.

$$\text{log2}\left({\varvec{I}\varvec{P}}_{Cy5\;normalized\;intensity}\right)=\text{log2}\left({\varvec{I}\varvec{P}}_{Cy5\;raw}\right)-\text{Average}\left[\text{log2}\left({\varvec{I}\varvec{P}}_{spike-in\_Cy5\;raw}\right)\right]$$

$$\text{log2}\left({\varvec{S}\varvec{u}\varvec{p}}_{Cy3\;normalized\;intensity}\right)=\text{log2}\left({\varvec{S}\varvec{u}\varvec{p}}_{Cy3\;raw}\right)-\text{Average}\left[\text{log2}\left({\varvec{S}\varvec{u}\varvec{p}}_{spike-in\_Cy3\;raw}\right)\right]$$

The “m6A quantity” was calculated for the m6A methylation amount of each transcript based on the IP (Cy5-labelled) normalized intensities.

$$\text{Sample m6A quantity}= \text{Sample}\;{\varvec{I}\varvec{P}}_{Cy5\;normalized\;intensity}$$

Postscript: Raw intensities of IP (Cy5-labelled) were normalized by average of log2-scaled Spike-in RNA intensities.

$$\text{log2}\left({\varvec{I}\varvec{P}}_{Cy5\;normalized\;intensity}\right)=\text{log2}\left({\varvec{I}\varvec{P}}_{Cy5\;raw}\right)-\text{Average}\left[\text{log2}\left({\varvec{I}\varvec{P}}_{spike-in\_Cy5\;raw}\right)\right]$$

### Cells and culture

OSCC cells (SCC25 cells) were provided by ATCC (American Type Culture Collection, Manassas, VA, USA) and cultured in DMEM Medium supplemented with fetal bovine serum (FBS, 10%), 100 U/ml penicillin, 100 µg/ml streptomycin. Normal cells (Human Oral Keratinocytes, HOK, Catalog No. 2610, ScienCell) were provided by ScienCell (San Diego, California, USA). HOK cells were cultured in Oral Keratinocyte Medium (OKM, Cat. No. 2611, ScienCell) recommended by ScienCell in vitro. Cells were incubated in a 37 °C humidified incubator with 5% CO_2_.

### m^6^A methylated RNA immunoprecipitation sequencing (MeRIP-seq)

The total RNA was extracted from cell samples, and then divided into two groups, including Input control sample and immunoprecipitation (IP) sample. The RNAs were firstly spliced into ~ 100nt fragments. IP samples provide unbiased measurements of methylated RNA fragments with specific m^6^A antibodies. Meanwhile, the Input control sample reflected the abundance of basic RNA enrichment. The reference genome was hg38 gencode. Through library construction, high-throughput sequencing and bioinformatics analysis, mapping of whole transcriptome m^6^A location was generated by Jiayin Biotechnology Ltd. (Shanghai, China).

### Statistical analysis

Differentially m^6^A-methylated RNAs between two comparison groups were identified by filtering with the fold change (> 1.5 fold) and statistical significance (*p*-value < 0.05) thresholds. Data were shown as means ± standard deviation (SD).

## Supplementary Information

**Additional file 1.**

**Additional file 2.**

## Data Availability

The raw data that support the findings of this study has been deposited into NCBI Gene Expression Omnibus (GEO): accession number GSE198105. Researchers may view the GSE198105 study at: https://www.ncbi.nlm.nih.gov/geo/query/acc.cgi?acc=GSE198105, and GSE197457 (https://www.ncbi.nlm.nih.gov/geo/query/acc.cgi?acc=GSE197457).

## Abbreviations

m^6^A: N6-methyladenosine
circRNA: circular RNA
