# Supplementary material for: High-throughput microarray reveals the epitranscriptome-wide landscape of m6A-modified circRNA in oral squamous cell carcinoma
Source: BMC Genomics. 2022 Aug 23;23:611. doi: 10.1186/s12864-022-08806-z (PMC9400228; doi:10.1186/s12864-022-08806-z)
Supplement: Supplementary file 1 — Additional file 1. [file 12864_2022_8806_MOESM1_ESM.docx]

**Supplementary Figure S1.**


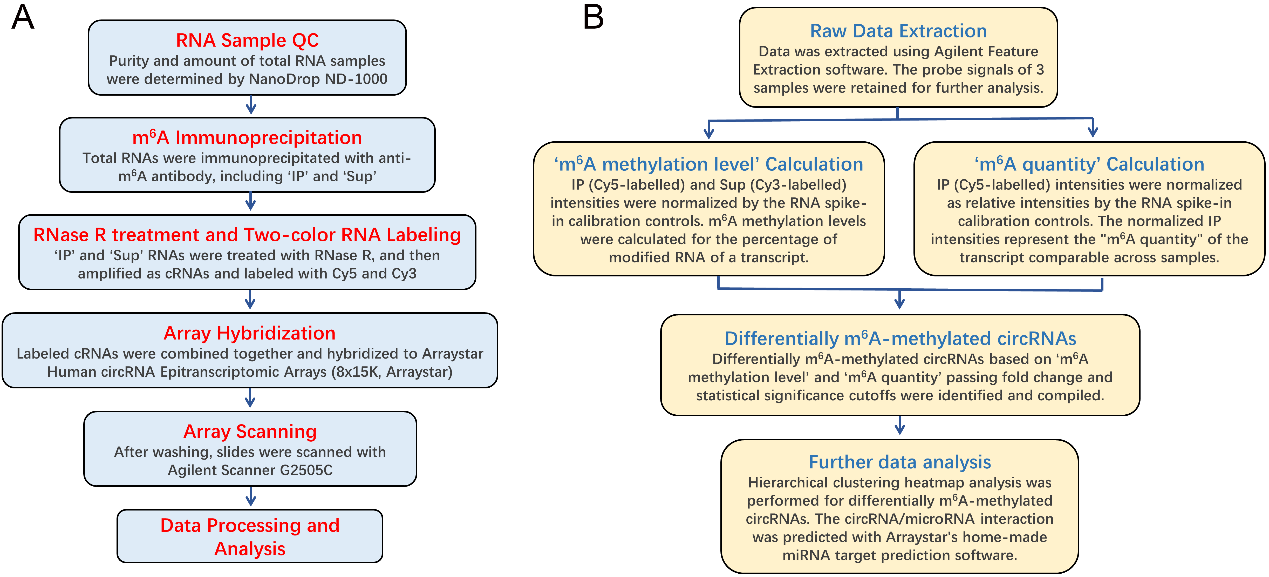


**Supplementary Figure S1. Workflow of m^6^A-circRNA epitranscriptomic microarray analysis.** (A) Graphical representation displayed the RNA extraction, quality control (QC), library construction from OSCC cells (3 independent samples, SCC25 cells) and normal cells (3 independent samples, HOK cells). ‘IP’ group (immunoprecipitated RNAs) indicated the m^6^A-modified RNAs eluted from the immunoprecipitated magnetic beads. ‘Sup’ group (supernatant unmodified RNAs) indicated the unmodified RNAs recovered from the supernatant. (B) Raw data analysis was performed from two aspects, including m^6^A methylation level and m^6^A quantity.

**Supplementary Figure S2.**

**ceRNA network of potential circRNA-****miRNA-mRNA in OSCC**

Given that circRNAs could act as miRNA sponge to harbor their downstream miRNAs, thereby releasing the fettered target mRNAs to modulate the cancer progression. The regulation pattern was regarded as competing endogenous RNA (ceRNA). Here, our lab utilized the m^6^A-circRNA epitranscriptomic microarray analysis to analyze the potential circRNA-miRNA-mRNA in OSCC. A great number pairs of circRNA-mRNA and miRNA-mRNA were found in the results. For a better exhibition, we chose two circRNA (hsa_circRNA_101017, hsa_circRNA_406747) to show the ceRNA network in OSCC (**Figure S2**).


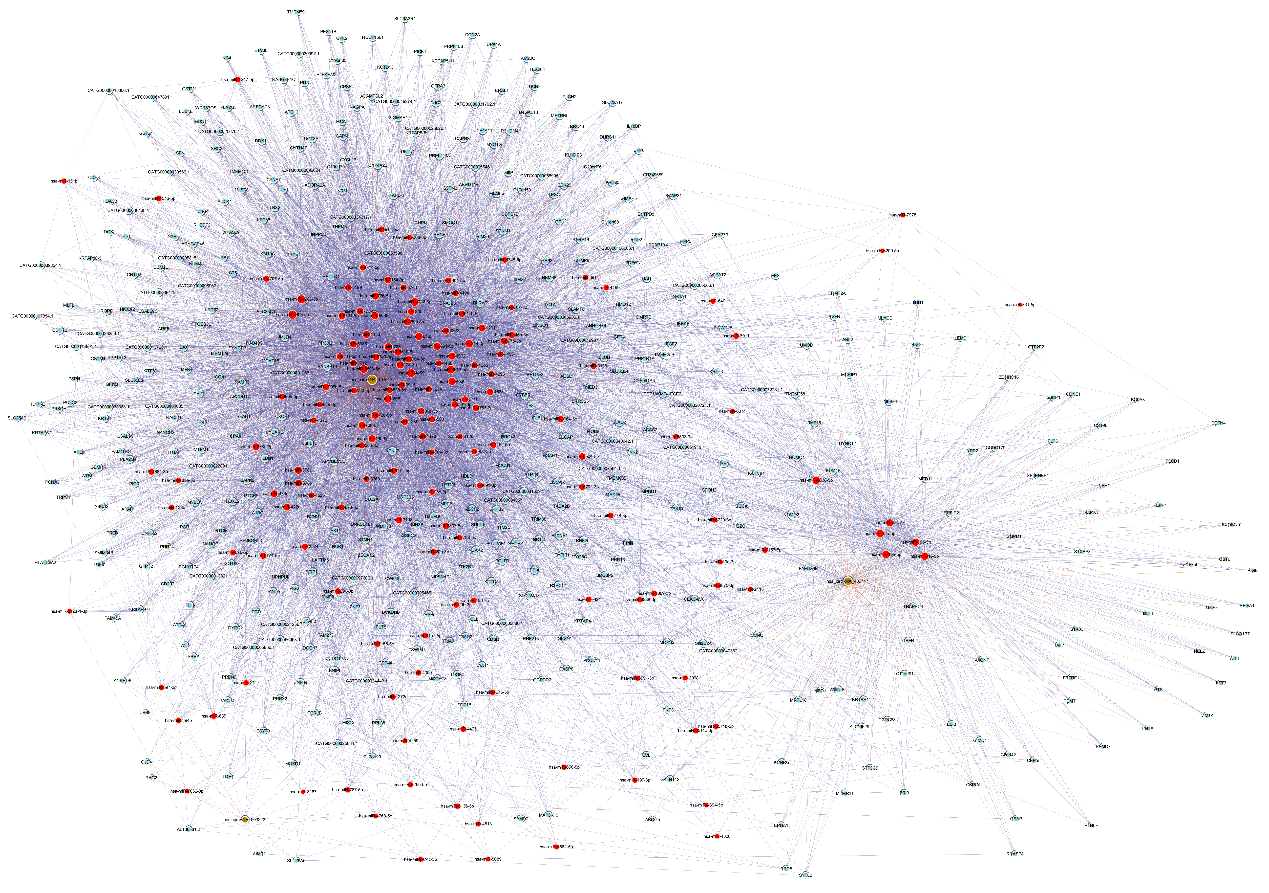


**Figure S2. ceRNA network of potential circRNA-miRNA-mRNA in OSCC.** A great number pairs of circRNA-mRNA and miRNA-mRNA were found in the results. For a better exhibition, we chose two circRNA (hsa_circRNA_101017, hsa_circRNA_406747) to show the ceRNA network in OSCC.
