# Supplementary material for: High-throughput microarray reveals the epitranscriptome-wide landscape of m6A-modified circRNA in oral squamous cell carcinoma
Source: BMC Genomics. 2022 Aug 23;23:611. doi: 10.1186/s12864-022-08806-z (PMC9400228; doi:10.1186/s12864-022-08806-z)
Supplement: Supplementary file 2 — Additional file 2. [file 12864_2022_8806_MOESM2_ESM.docx]

**Highlights**

1. Here, we report an epitranscriptome-wide mapping of m6A-modified circRNAs (m6A-circRNA) in oral squamous cell carcinoma (OSCC).

2. Utilizing the data of MeRIP-seq and m6A-circRNAs epitranscriptomic microarray analysis, we found that m6A-circRNAs exhibited their particular modification style in OSCC, which was independent of m6A-mRNA.

3. m6A modification on circRNAs frequently occurred on the long exons in the front part of the coding sequence (CDS), which was distinct from m6A-mRNA that in 3’-UTR or stop codon.

4. Our work preliminarily demonstrates the traits of m6A-circRNAs and probed into the association of m6A-circRNAs and m6A-mRNA.
